# Are Machine Learning Algorithms More Accurate in Predicting Vegetable and Fruit Consumption Than Traditional Statistical Models? An Exploratory Analysis

**DOI:** 10.3389/fnut.2022.740898

**Published:** 2022-02-17

**Authors:** Mélina Côté, Mazid Abiodoun Osseni, Didier Brassard, Élise Carbonneau, Julie Robitaille, Marie-Claude Vohl, Simone Lemieux, François Laviolette, Benoît Lamarche

**Affiliations:** ^1^Centre Nutrition, santé et société (NUTRISS), Institut sur la nutrition et les aliments fonctionnels de l'Université Laval (INAF), Université Laval, Québec, QC, Canada; ^2^École de nutrition, Université Laval, Québec, QC, Canada; ^3^Centre de recherche en données massives (CRDM), Université Laval, Québec, QC, Canada; ^4^Groupe de recherche en apprentissage automatique de l'Université Laval (GRAAL), Université Laval, Québec, QC, Canada

**Keywords:** artificial intelligence, machine learning, statistical models, nutrition, prediction, dietary behaviour

## Abstract

Machine learning (ML) algorithms may help better understand the complex interactions among factors that influence dietary choices and behaviors. The aim of this study was to explore whether ML algorithms are more accurate than traditional statistical models in predicting vegetable and fruit (VF) consumption. A large array of features (2,452 features from 525 variables) encompassing individual and environmental information related to dietary habits and food choices in a sample of 1,147 French-speaking adult men and women was used for the purpose of this study. Adequate VF consumption, which was defined as 5 servings/d or more, was measured by averaging data from three web-based 24 h recalls and used as the outcome to predict. Nine classification ML algorithms were compared to two traditional statistical predictive models, logistic regression and penalized regression (Lasso). The performance of the predictive ML algorithms was tested after the implementation of adjustments, including normalizing the data, as well as in a series of sensitivity analyses such as using VF consumption obtained from a web-based food frequency questionnaire (wFFQ) and applying a feature selection algorithm in an attempt to reduce overfitting. Logistic regression and Lasso predicted adequate VF consumption with an accuracy of 0.64 (95% confidence interval [CI]: 0.58–0.70) and 0.64 (95%CI: 0.60–0.68) respectively. Among the ML algorithms tested, the most accurate algorithms to predict adequate VF consumption were the support vector machine (SVM) with either a radial basis kernel or a sigmoid kernel, both with an accuracy of 0.65 (95%CI: 0.59–0.71). The least accurate ML algorithm was the SVM with a linear kernel with an accuracy of 0.55 (95%CI: 0.49–0.61). Using dietary intake data from the wFFQ and applying a feature selection algorithm had little to no impact on the performance of the algorithms. In summary, ML algorithms and traditional statistical models predicted adequate VF consumption with similar accuracies among adults. These results suggest that additional research is needed to explore further the true potential of ML in predicting dietary behaviours that are determined by complex interactions among several individual, social and environmental factors.

## Introduction

Artificial intelligence (AI) has become prominent in healthcare research, particularly in precision medicine, for assessing disease risk, identifying potential complications or selection of treatment (1–3). For instance, machine learning (ML) algorithms have been used to predict risk of different chronic diseases and often, ML algorithms have outperformed traditional statistical models (4–8). Among others, ML algorithms can account for non-linear and high dimensional relationships, which may lead to better predictive performances. The availability of voluminous and rich datasets, such as Electronic Health Records, longitudinal data and omics data, has also accelerated the use of ML algorithms and other AI methods in health research (9–12).

The rapid and successful progress in precision medicine based on ML suggests promising applications in other fields including public health nutrition, where important amounts of data are already available (13), yet largely unexploited. Indeed, healthy eating is the sum of interactions among several complex behaviours and individual, social and environmental factors. To that extent, ML algorithms may help achieve a more comprehensive understanding of factors that are associated with, influence or determine the quality of the diet at the individual or population level. This is an important area to explore because low quality diets are responsible for half of the deaths associated with chronic diseases globally, which is more than any other risk factors, including smoking (14). Yet, despite several public health efforts and policies, adhering to healthy eating remains a challenge.

It needs to be stressed that the advantage of using ML algorithms over traditional statistical models to predict a health outcome has not always been observed (15–19). For instance, a systematic review found no evidence that ML algorithms had better accuracy than logistic regression for clinical prediction modeling (15). Another study also found no clear difference in performance between regression models, including logistic regression and lasso regression, and ML algorithms for prognostication of traumatic brain injury (16). Similarly, a study demonstrated that logistic regression performed equally to ML algorithms in predicting the risk of multiple chronic diseases (18). Exploring potential applications of ML algorithms to the broad field of nutrition is therefore timely as we know little about their advantage over traditional statistical models (9).

To the best of our knowledge, the present study is one of the first to compare ML algorithms to traditional statistical models to predict a dietary behavior. Specifically, the aim of this study was to explore and compare the performance metrics of ML algorithms and traditional statistical models to predict a simple healthy dietary behavior, i.e., adequate vegetable and fruit (VF) consumption, using a large array of individual, social and environmental features. We hypothesized that ML algorithms are more accurate than traditional statistical models in predicting adequate VF consumption. We stress that this analysis was not intended to provide a definitive predictive model of adequate VF consumption.

## Materials and Methods

### Study Population

Data used for these analyses are from the PREDISE (PRÉDicteurs Individuels, Sociaux et Environnementaux) study, a web-based study which purpose is to investigate how individual, social and environmental factors are associated to adherence to healthy eating recommendations among French-speaking adults from the province of Québec, Canada. The PREDISE study design and methodology have been previously detailed elsewhere (20). Briefly, participants aged between 18 and 65 years of age were recruited between August 2015 and April 2017 using random digit dialing in five different administrative regions in the province of Québec. Participants completed online questionnaires regarding individual, social and environmental factors, three web-based 24 h dietary recalls and a web-based food frequency questionnaire (wFFQ). The complete list of questionnaires is provided in the Supplementary Table 1. Once all questionnaires had been completed, participants were invited to their regional's research center for clinical assessment (anthropometric measurements and blood sampling). The project was conducted in accordance with the Declaration of Helsinki and was approved by the Research Ethics Committees of Université Laval (ethics number: 2014-271), Centre hospitalier universitaire de Sherbrooke (ethics number: MP-31-2015-997), Montreal Clinical Research Institute (ethics number: 2015-02), and Université du Québec à Trois-Rivières (ethics number: 15-2009-07.13).

### Assessment of Vegetable and Fruit Intake

Participants from the PREDISE study were invited by email on three randomly selected separate unannounced days to complete a self-administered 24-h web recall, the R24W. The development and validation of the R24W has been detailed elsewhere (21–24). Of the 1147 participants, 1083 participants (94.4%) completed all three recalls, 34 participants (3%) completed two recalls and 30 participants (2.6%) completed only one recall. VF intake (in servings/day), as defined in Canada's Food Guide 2007 (25), was calculated by averaging intakes from all recalls available. Participants of the PREDISE study were also invited to complete a self-administered wFFQ composed of 136 questions to reflect dietary intake over the past 30 days. The wFFQ has been previously validated for the studied population (26).

### Predictors and Outcome Variable

The set of predictor variables and their corresponding features were derived from all questions and scores from all questionnaires listed in Supplementary Table 1. A variable represented a question in a given questionnaire, while its corresponding features reflected the transformed variable, for example, dummy variables for each response to that question. Data from the clinical assessment, which includes serum cholesterol, triglycerides, HDL-cholesterol, fasting blood glucose and insulin concentrations, systolic and diastolic blood pressures were also considered as features in each model and algorithm. Age, sex, measured height, measured weight, body mass index, body fat percentage and waist circumference were considered as features in all models and algorithms. Questions that had been completed by <70% of the participants were excluded, resulting in 525 predictor variables. Missing data for continuous features were imputed using the study population averages for each feature. The categorical variables were dummy coded with a specific binary code for missing data. Once categorical variables were dummy coded, total number of predictor features included on all models and algorithms was 2,452.

The outcome predicted (classes) was VF intake dichotomized as adequate/inadequate, based on the population target in Québec of 5 or more servings/d (27). Specifically, the two classes were 1- adequate VF consumption, corresponding to 5 or more servings/d and 2- inadequate VF consumption, corresponding to less than 5 servings/d.

### Data Modelling

Logistic regression (LR) (28) and penalized regression (Lasso) (29) were considered the reference classification/predictive models while nine commonly known supervised ML classification algorithms were applied: decision tree (DT) (30), random forest (RF) (31), set-covering machine (SCM) (32), support vector machines (SVM) (33) with different kernels (linear, polynomial, radial basis, sigmoid), k-nearest neighbour (KNN) (34) and Adaboost (35). Table 1 provides a short description of each classification model and algorithm. ML algorithms have different hyperparameters to be optimized to achieve the best predictive models possible. The hyperparameters were selected using five-fold cross-validation (Supplementary Table 2). Data was split in two non-overlapping sets, the train set containing 80% of the sample to develop the models and algorithms and the test set using 20% of the sample to evaluate model and algorithm performances. As part of the iterative process needed to maximize the performance of the classification algorithms, the distribution of continuous data was rescaled between 0 and 1 to normalize the data across all features. As shown in Supplementary Figure 1, the accuracy of the LR, KNN, SVM with linear kernel, radial basis kernel and sigmoid kernel algorithms to predict adequate VF consumption was improved when using normalized compared to non-normalized data. Normalizing the data had little impact on the accuracy of the Lasso, DT, RF, SCM, and Adaboost algorithms. The SVM with polynomial kernel algorithm was the only ML algorithm for which data normalization decreased accuracy. Subsequent analyses were therefore undertaken using normalized data for continuous features. All analytical steps of model development (normalizing the data, developing/training and testing) were bootstrapped 15 times to generate measurement errors and hence 95% confidence intervals (95%CI) for each performance metric. The models and algorithms were compared using common metrics in a ML classification framework problem: accuracy, area under the receiver operating characteristic curve (AUROC), precision (positive predictive value), recall (sensitivity), and F1 score (Table 2). Finally, the list of discriminant features retained in the LR, Lasso, DT, RF, SCM, SVM linear and Adaboost models and algorithms were compared to verify any similarities or differences. The discriminant analysis was conducted by identifying the 10 features with the highest coefficients for LR, Lasso and SVM linear. Gini feature importance was used for the discriminant analysis of the RF and Adaboost algorithms, and Entropy importance was used for the DT algorithm. All features retained by the SCM corresponded to the discriminant features for this algorithm. KNN does not rank features based on importance and SVM (polynomial, radial basis and sigmoid) are uninterpretable. Thus, data from these algorithms were not included in the discriminant analysis.

**Table 1:** Model and algorithm description.

**Classification models and algorithms**	**Machine learning algorithm**	**Description**
Logistic regression (28)	Not typically	Model that calculates the probability of belonging to one of two classes (if outcome is binary) by computing the logit function of the combination of weighted input features. The weights are estimated using maximum-likelihood estimation.
Lasso (Least absolute shrinkage and selection operator) (29)	Not typically	Model that uses feature selection and shrinkage to reduce the number of features for classification purposes. The coefficients of features that are useless to the model are shrunk to zero.
Decision tree (30)	Yes	Algorithm with a flowchart-like structure that makes predictions by learning decision rules. Each node represents an input feature, each branch represents a decision rule and each leaf represents a prediction. The top of the tree represents the best predictor and input features are compared until a leaf node is reached.
Random forest (31)	Yes	Algorithm that generates a large ensemble of decision trees with bootstrapped samples of the data. The predicted class is then determined by averaging the estimated outcome variable of each decision tree.
Set-covering machine (32)	Yes	Algorithm that learns a conjunction or disjunction of rules to find a decision function with the smallest number of rules.
Support vector machine (33)	Yes	Algorithm that attempts sorting the data between two classes with a hyperplane. The hyperplane can either be a linear, a polynomial, a radial basis or a sigmoid function and is determined using only the points closest to the hyperplane.
K-nearest neighbor (34)	Yes	Algorithm that assumes that close data points are similar. The class in which a new data point belongs is determined according to the shared characteristics of a pre-determined number of closest points.
Adaboost (35)	Yes	Algorithm that fits a classifier (ex: decision tree) to the dataset and then adjusts the weights of the incorrectly classified data points, forcing the algorithm to focus on the data that is more difficult to classify.

**Table 2:** Predictive metrics and corresponding equations.

**Metric**	**Equation**
Accuracy	(True positives + True negatives)/Total Sample
Precision (positive predictive value)	True positives/(False positives+ True positives)
Recall (sensitivity)	True positives/(True positives + False negatives)
F1 score	2 × (Precision * Recall)/(Precision + Recall)

A series of sensitivity analyses were performed to examine if and how particular aspects of the data differentially influenced the performance of traditional statistical models and ML-based classification algorithms. First, the models and algorithms were tested using VF intake data from the wFFQ. Unlike 24-hr recalls, which measure short term consumption, food frequency questionnaires measure longer term consumption of foods, yielding data that are less influenced by within-person (random) errors (i.e., day-to-day variability in intakes) than data derived from the R24W. For that purpose, VF consumption from the wFFQ was also dichotomized using the 5 servings/d cut-off. Second, other diet-related features obtained from the R24W were included in the analyses, including Canada's Food Guide 2007 servings of grain products, milk and alternatives, meat and alternatives, as well as components of the Canadian Healthy Eating Index (C-HEI) (36) other than the VF component and the C-HEI score itself. This was undertaken to validate the increase in accuracy when such features are considered because they correlate closely with VF consumption. Third, to attempt to overcome overfitting, a feature selection algorithm was applied to reduce the number of features to 5, 10, and 50 features. The feature selection algorithm selects a pre-determined number of best features based on univariate statistical tests. All analyses apart from the bootstrapped results were conducted with the same random state, ensuring that the train and test datasets were identical from one model and algorithm to the other. All analyses were carried out in Python 3.7. Preprocessing, statistical models and ML algorithms, feature selection (Select K best) and metrics were computed using scikit-learn packages. Execution time of algorithms varied between 5 secs and 7 mins (Supplementary Table 3).

## Results

Table 3 shows characteristics of the 1,147 participants (572 women, 575 men) included in the present study. The majority of participants had a university degree and were Caucasian. The mean (±standard deviation) VF consumption evaluated by the R24W in the sample was 5.5 ± 3.1 servings/d (interquartile range = 4.0), with 52.3% of participants consuming 5 or more servings/d. The mean VF consumption evaluated by the wFFQ was 7.6 ± 5.0 (interquartile range = 4.8) with 67.6% consuming 5 or more servings/d.

**Table 3:** Sociodemographic characteristics of the French-speaking adults from Quebec, Canada (*N* = 1,147).

**Characteristics**	***n* (%)**
Age group, y	
18–34	432 (37.7)
35–49	330 (28.8)
50–65	385 (33.5)
Sex	
Female	572 (50)
Male	575 (50)
Education	
High school or less	270 (23.5)
CEGEP^*^	332 (29.0)
University	485 (42.3)
Missing information	60 (5.2)
Household income, CAD $	
<30, 000	163 (14.2)
30, 000 to <60, 000	281 (24.5)
60, 000 to <90, 000	196 (17.1)
≥ 90, 000	348 (30.3)
Missing information	159 (13.9)
Ethnicity	
Caucasian	997 (86.9)
Arabic	25 (2.2)
Hispanic	19 (1.7)
Other	32 (2.8)
Missing information	74 (6.4)
Administrative region	
Capitale-Nationale/Chaudière-Appalaches	416 (36.3)
Estrie	121 (10.5)
Mauricie	98 (8.5)
Montreal	410 (35.8)
Saguenay-Lac-St-Jean	102 (8.9)

* *CEGEP is a preuniversity and technical college institution specific to the Quebec educational system*.

Table 4 presents the metrics of all models and algorithms predicting adequate VF consumption (≥5 servings/d) based on normalized data among all participants. There are no significant differences in accuracy between models and algorithms and no important differences for other performance metrics, including AUROC. When predicting inadequate VF consumption (<5 servings/d) instead of adequate VF consumption, results were essentially similar i.e., there were no differences in performance between traditional statistical models and ML algorithms (not shown).

**Table 4:** Performance metrics of two traditional statistical models and nine machine learning algorithms to predict adequate vegetable and fruit (VF) consumption based on dietary intake data obtained from web-based 24-hr recalls (R24W) among1147 French-speaking adults from Québec, Canada.

		Algorithms^*^										
	**Performance** **metric**	**Traditional statistical** **models (reference)**		**DT**	**RF**	**SCM**	**SVM**				**KNN**	**Adaboost**
		**LR**	**Lasso**				**Linear**	**Polynomial**	**Radial** **basis**	**Sigmoid**		
	**Accuracy TRAIN (95%CI)**	0.75 (0.73–0.77)	0.76 (0.74–0.78)	0.66 (0.58–0.74)	0.94 (0.82–1.06)	0.64 (0.60–0.68)	1.00 (1.00–1.00)	0.80 (0.64–0.96)	0.86 (0.72–1.00)	0.75 (0.73–0.77)	0.73 (0.48–0.98)	0.80 (0.68–0.92)
	**Accuracy TEST (95%CI)**	0.64 (0.58–0.70)	0.64 (0.60–0.68)	0.62 (0.58–0.74)	0.64 (0.56–0.72)	0.62 (0.54–0.70)	0.55 (0.49–0.61)	0.64 (0.58–0.70)	0.65 (0.59–0.71)	0.65 (0.59–0.71)	0.58 (0.50–0.66)	0.60 (0.56–0.64)
**Predicting adequate VF consumption (≥5 servings/d)**	**AUROC (95%CI)**	0.64 (0.58–0.70)	0.68 (0.62–0.74)	0.62 (0.54–0.70)	0.63 (0.57–0.69)	0.62 (0.56–0.68)	0.55 (0.49–0.61)	0.64 (0.58–0.70)	0.65 (0.59–0.71)	0.64 (0.58–0.70)	0.57 (0.51–0.63)	0.60 (0.56–0.64)
	**Positive predictive value**^a^ **(95%CI)**	0.65 (0.57–0.73)	0.65 (0.57–0.73)	0.63 (0.53–0.73)	0.63 (0.53–0.73)	0.63 (0.53–0.73)	0.57 (0.49–0.65)	0.64 (0.56–0.72)	0.65 (0.57–0.73)	0.64 (0.56–0.72)	0.58 (0.50–0.66)	0.61 (0.55–0.67)
	**Sensitivity**^b^ **(95%CI)**	0.68 (0.58–0.78)	0.68 (0.60–0.76)	0.66 (0.52–0.80)	0.73 (0.65–0.81)	0.67 (0.53–0.81)	0.58 (0.50–0.66)	0.72 (0.62–0.82)	0.72 (0.64–0.80)	0.74 (0.68–0.80)	0.69 (0.57–0.81)	0.61 (0.51–0.71)
	**F1 Score (95%CI)**	0.66 (0.60–0.72)	0.66 (0.60–0.72)	0.64 (0.56–0.72)	0.68 (0.62–0.74)	0.65 (0.57–0.73)	0.58 (0.52–0.64)	0.67 (0.59–0.75)	0.68 (0.62–0.74)	0.69 (0.63–0.75)	0.63 (0.55–0.71)	0.61 (0.55–0.67)

* *15 bootstrap resamples were used to estimate 95%CI*.

*LR, logistic regression; DT, decision tree; RF, random forest; SCM, set-covering machine; SVM, support vector machine; KNN, k-nearest neighbors*.

a *Positive predictive value, also referred to as precision*.

b *Sensitivity, also referred to as recall*.

**Figure 1:**
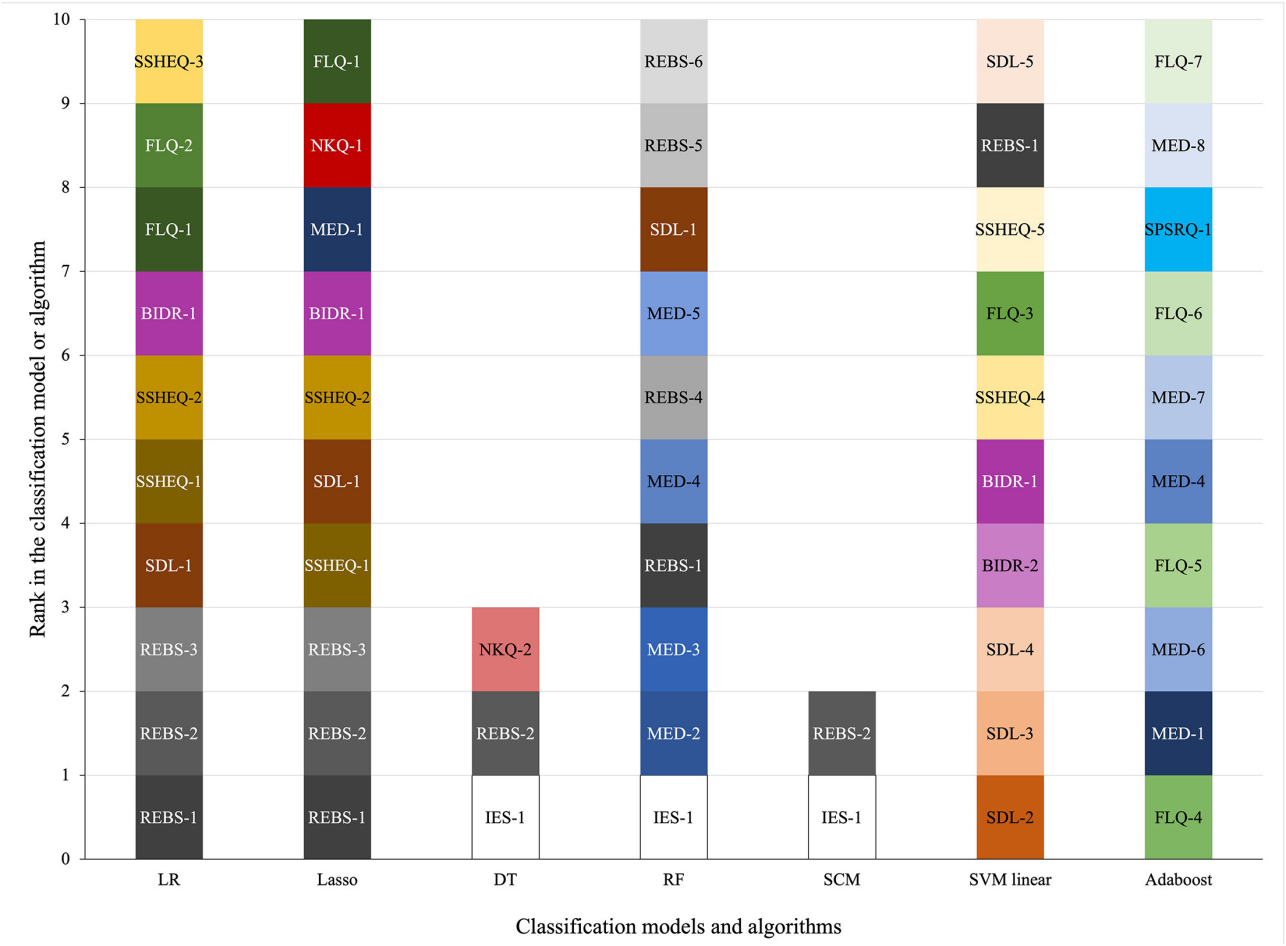
Discriminant features retained in the logistic regression **(LR)** and Lasso models and in the decision tree **(DT)**, random forest **(RF)**, set-covering machine **(SCM)**, support vector machine **(SVM)** with a linear kernel and Adaboost machine learning algorithms to predict adequate vegetable and fruit consumption. Features are colour-coded according to the questionnaire to which they belong; different shades within a given color indicate that more than one feature of a questionnaire was retained; numbers indicate the rank of a given question from a given questionnaire retained in the model or algorithm. REBS, Regulation of Eating Behaviour Scale; SDL, Socioeconomic and demographic factors, eating and lifestyle habits; SSHEQ, Social support for healthy eating questionnaire; BIDR, Balanced inventory of desirable responding; FLQ, Food liking questionnaire; MED, Medical questionnaire; NKQ, Nutrition knowledge questionnaire; IES, Intuitive eating scale; SPSRQ, Sensitivity to punishment and sensitivity to reward questionnaire.

Figure 1 presents the top discriminant features included in seven of the classification models and algorithms. Discriminant features are colour-coded for illustrative purposes to allow rapid visual comparison. Figure 1 shows that the discriminant features predicting adequate VF consumption are inconsistent across models and algorithms. While the traditional classification models LR and Lasso shared eight top discriminant features, there is little coherence between the discriminant features of the five ML algorithms. No single feature was included as a top discriminant feature in all seven models and algorithms.

As shown in Table 5, traditional statistical models and ML classification algorithms also showed comparable performance metrics using VF consumption data obtained from the wFFQ, which is less prone to within-individual variability than data from a 24-h recall such as the R24W. Of note, the majority of ML algorithms in this sensitivity analysis predicted adequate VF consumption with a slightly higher accuracy when using data from the wFFQ (accuracy values ranging between 0.63 to 0.70) than when using data from the R24W (accuracy values ranging between 0.55 to 0.65, Table 4). Positive predictive values, sensitivity and F1 scores were also higher when using intake data from the wFFQ compared to data from the R24W. AUROC values for all models and algorithms were lower when using data from the wFFQ compared to data from the R24W, except for the Lasso model for which the AUROC value slightly increased.

**Table 5:** Performance metrics of two traditional models and nine machine learning algorithms to predict adequate vegetable and fruit (VF) consumption based on dietary intake data obtained from a web-based food frequency questionnaire (wFFQ) among1147 French-speaking adults from Québec, Canada.

		Algorithms^*^										
	**Performance** **metric**	**Traditional statistical** **models (reference)**		**DT**	**RF**	**SCM**	**SVM**				**KNN**	**Adaboost**
		**LR**	**Lasso**				**Linear**	**Polynomial**	**Radial** **basis**	**Sigmoid**		
	**Accuracy TRAIN (95%CI)**	0.76 (0.74–0.78)	0.78 (0.76–0.80)	0.69 (0.63–0.75)	0.91 (0.85–0.97)	0.69 (0.67–0.71)	1.00 (1.00–1.00)	0.96 (0.78–1.00)	0.90 (0.82–0.98)	0.76 (0.52–1.00)	0.92 (0.67–1.00)	0.82 (0.72–0.92)
	**Accuracy TEST (95%CI)**	0.70 (0.62–0.78)	0.70 (0.62–0.78)	0.67 (0.61–0.73)	0.68 (0.62–0.74)	0.66 (0.60–0.72)	0.63 (0.59–0.67)	0.67 (0.61–0.73)	0.69 (0.65–0.73)	0.66 (0.60–0.72)	0.67 (0.61–0.73)	0.67 (0.61–0.73)
**Predicting adequate VF consumption (≥5 servings/d)**	**AUROC (95%CI)**	0.59 (0.53–0.65)	0.72 (0.64–0.80)	0.52 (0.44–0.60)	0.52 (0.50–0.54)	0.51 (0.47–0.55)	0.58 (0.54–0.62)	0.57 (0.49–0.65)	0.57 (0.51–0.63)	0.52 (0.44–0.60)	0.55 (0.49–0.61)	0.61 (0.55–0.67)
	**Positive predictive value**^a^ **(95%CI)**	0.72 (0.64–0.80)	0.72 (0.64–0.80)	0.68 (0.60–0.76)	0.68 (0.62–0.74)	0.68 (0.60–0.76)	0.72 (0.66–0.78)	0.71 (0.65–0.77)	0.71 (0.63–0.79)	0.69 (0.61–0.77)	0.70 (0.64–0.76)	0.74 (0.68–0.80)
	**Sensitivity**^b^ **(95%CI)**	0.91 (0.87–0.95)	0.90 (0.84–0.96)	0.95 (0.75–1.00)	0.99 (0.97–1.00)	0.96 (0.84–1.00)	0.74 (0.66–0.82)	0.86 (0.74–0.98)	0.90 (0.80–1.00)	0.93 (0.71–1.00)	0.90 (0.82–0.98)	0.80 (0.74–0.86)
	**F1 Score (95%CI)**	0.80 (0.74–0.86)	0.80 (0.74–0.86)	0.79 (0.73–0.85)	0.81 (0.77–0.85)	0.79 (0.75–0.83)	0.73 (0.69–0.77)	0.78 (0.72–0.84)	0.79 (0.75–0.83)	0.78 (0.72–0.84)	0.78 (0.74–0.82)	0.77 (0.73–0.81)

* *15 bootstrap resamples were used to estimate 95%CI*.

*LR, logistic regression; DT, decision tree; RF, random forest; SCM, set-covering machine; SVM, support vector machine; KNN, k-nearest neighbors*.

a *Positive predictive value, also referred to as precision*.

b *Sensitivity, also referred to as recall*.

**Figure 2:**
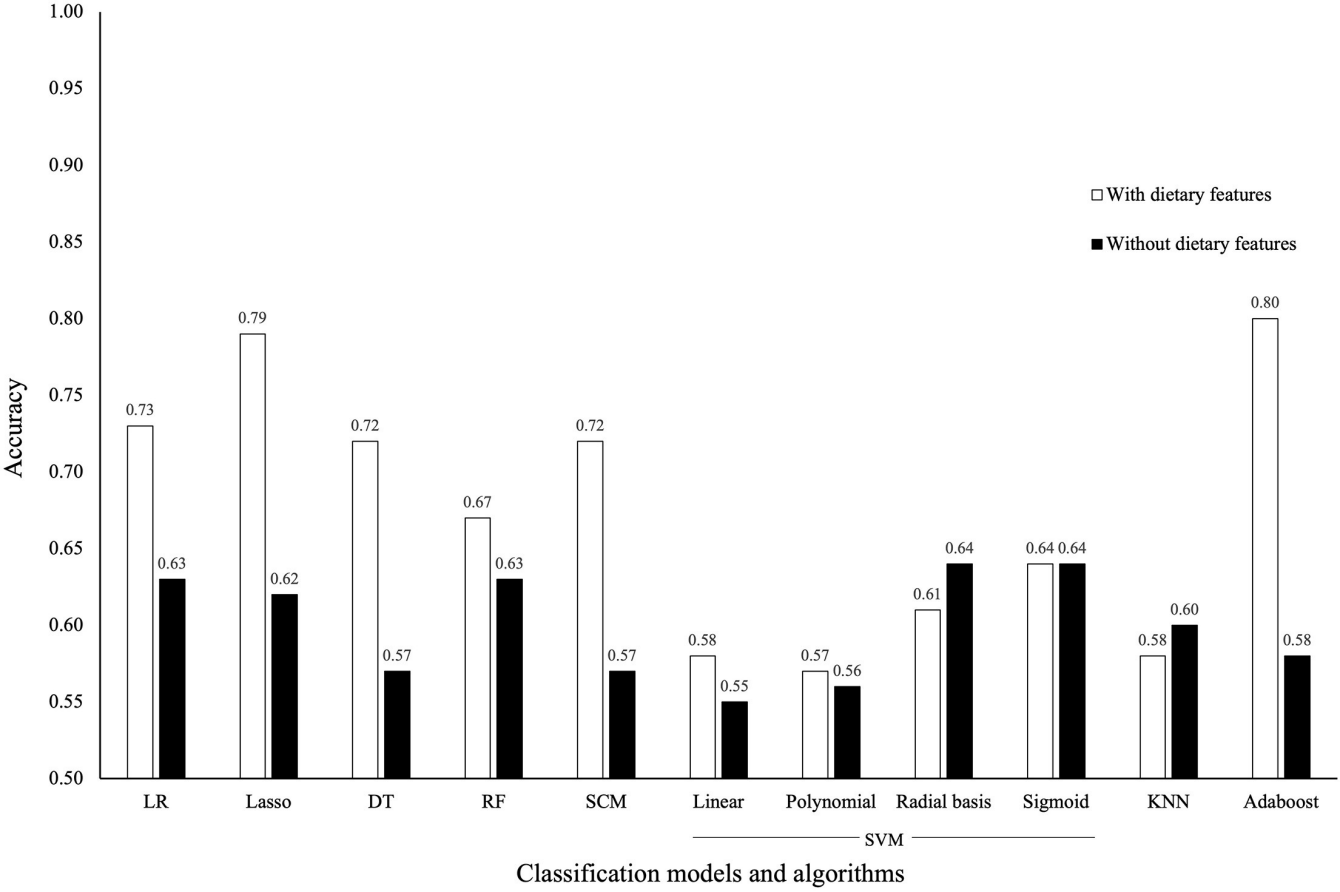
Comparing the accuracy of traditional statistical models and machine learning algorithms to predict adequate vegetable and fruit **(VF)** consumption when other dietary intake features are included in addition to the 2452 features originally included. These are servings of grain products, milk and alternatives, meat and alternatives, as well as components of the Canadian Healthy Eating Index **(C-HEI)** other than the VF component and the C-HEI score itself. LR, logistic regression; DT, decision tree; RF, random forest; SCM, set-covering machine; SVM, support vector machine; KNN, k-nearest neighbor.

The accuracy of traditional statistical models and of ML classification algorithms increased when dietary features known to be correlated with VF consumption were included in the analyses (Figure 2). Other performance metrics are reported in Supplementary Table 4. Accuracy of the various ML classification algorithms in predicting adequate VF consumption was once again not superior to accuracy seen with traditional statistical models.

Finally, reducing the number of features to 5, 10, and 50 features with a feature selection algorithm attenuated overfitting for most models and algorithms, but had trivial and inconsistent impacts on accuracy values and other metrics (Supplementary Figure 2; Supplementary Table 5). Specifically, the accuracy of all models and algorithms remained low, and no differences were observed between traditional statistical models and ML algorithms when fewer features were used to predict an adequate VF consumption.

## Discussion

The successful use of ML in several healthcare fields suggests promising applications in the field of nutrition epidemiology and public health nutrition. However, the superiority and advantages of ML-based classification approaches compared with more traditional statistical approaches need to be evaluated, validated, and confirmed in all fields of application (3, 12, 37). The objective of this study was to compare the performance metrics of ML algorithms to those of more traditional statistical models in predicting a tangible and simple dietary behavior, i.e., VF consumption. The hypothesis that ML classification algorithms outperform traditional statistical classification models when predicting adequate VF consumption based on a wide spectrum of individual, social and environmental data was not supported by our experimental data.

This observation is not entirely inconsistent with data from previous studies in other fields of research, where ML classification algorithms and traditional statistical models performed equally. For example, ML classification algorithms such as SVM, neural network, RF, KNN and gradient boosting machine did not outperform traditional statistical models such as LR and penalized regression to predict the risk of type 1 and type 2 diabetes, traumatic brain injury, and fetal growth abnormalities (16, 17, 19). A study also demonstrated that LR outperformed ML classification algorithms when predicting chronic kidney diseases and diabetes in a prospective cohort study, LR being ranked among the best models when predicting the risk of cardiovascular disease and hypertension (18). In a systematic review, LR was shown to be equally accurate, if not better than ML classification algorithms (15).

This is somewhat incoherent with the paradigm that ML-based algorithms may be better suited for the exploitation of large and complex datasets than LR, which is considered more effective in situations where only a smaller number of features are available (9, 11, 18). In the present study, a rather large number of features were used. One possible reason explaining why ML classification algorithms did not outperform traditional statistical models in our study may be because VF consumption is a behavior that cannot be predicted with certainty. Consumption of VF was measured by averaging data from three 24-h recalls, which are known to be associated with random errors. Therefore, dichotomizing VF consumption is inevitably and intrinsically characterized by misclassification. Misclassification generated by random error in the measurement of VF consumption obviously limits one's ability to accurately predict adequate VF consumption. Studies in which ML classification algorithms performed better than traditional statistical models often predicted an outcome that was defined with a relatively high degree of certainty. For instance, the SVM and RF algorithms predicted survival rate after traumatic brain injuries as well as readmission after hospitalization for heart failure with greater accuracy than LR (7, 8). In the present study, accuracy of all models and algorithms increased when dietary intake data from the wFFQ were used in place of data from 24-h recalls. VF consumption measured over longer periods of time, such as with wFFQs, may be closer to the true usual intake, i.e., long-term average, and may therefore be more stable than when measured using average data from three 24-h recalls. However, this did not materialize into better performance metrics of ML classification algorithms compared to traditional statistical models. The fact that food frequency questionnaires are more prone to systematic error than 24-h recalls apparently did not negatively influence performance metrics of traditional statistical models and of ML classification algorithms.

Overall performances remained low for all classification models and algorithms tested in the present study. It is possible that the set of features did not contain domains of variables that may improve the prediction of adequate VF consumption. Indeed, the added value of large sets of data can be marginal if the relevant features are not included (3). This impacted the performance of ML classification algorithms as much as the traditional statistical models. The low accuracy may also be partly explained by the overfitting of certain models and algorithms. Overfitting occurs when the classification algorithms memorize observed patterns rather than learning relevant patterns (38). All models and algorithms, except DT and SCM, tended to slightly or substantially overfit despite normalizing the data from continuous features and adjusting hyperparameters to minimize overfitting and to optimize performances. Applying a feature selection algorithm to reduce the number of features included in the analyses lowered overfitting for all models and algorithms, but had little to no impact on accuracy. On the other hand, accuracy improved for the majority of the models and algorithms when dietary features closely associated with VF intake were included, but overall performance of ML classification algorithms and traditional statistical models remained comparable. The compelling observation is that the ML classification algorithms tested in the present study do not predict adequate VF consumption with more accuracy than traditional statistical models when using a large set of features.

Finally, features retained within the various classification models and algorithms to predict adequate VF consumption were inconsistent. Indeed, while LR and Lasso models included a relatively similar set of features, including for example factors from the *Regulation of Eating Behaviour Scale* questionnaire, ML algorithms were based on a completely different set of discriminant features such as, for example factors from the *Intuitive Eating Scale, Medical* or *Socioeconomic and demographic factors, eating and lifestyle* habits questionnaires. This suggests that different modelling approaches must always be tested in order to identify the most appropriate predictors for a given application. This also implies that multiple ML classification algorithms should always be compared because some may be better suited for use with nutrition-related data. Multicollinearity among a large set of related features can negatively affect the predictor selection, potentially reducing the face-validity and explainability of predictors included in the most models and algorithms (39, 40). Had our intent been to identify the predictors of adequate VF consumption, multicollinearity among features should have been considered. On the other hand, simulation studies have shown that multicollinearity has little to no impact on predictive performances (39, 40). Since the primary aim of this exploratory analysis was to compare predictive accuracy of different models and algorithms, multicollinearity did not have to be addressed. Future studies designed to identify discriminant features of adequate VF consumption or any other dietary behavior with traditional statistical models or with ML algorithms will need to consider multicollinearity.

### Strengths and Limitations

Our study lacked an external validation set. However, because our objective was to compare different classification approaches, and not to formally identify the features best predicting VF intake, this limitation is of less importance. Also, only one dietary behaviour was studied. Other dietary outcomes related to healthy diet recommendations, such as overall diet quality or eating with family members, may have yielded different results. Further research is therefore needed to evaluate the relevance and added value of using ML classification algorithms instead of traditional statistical models to predict other diet-related behaviours. Finally, our sample size remains small, which can affect the performance of ML algorithms (11). Our study also has the following strengths. To our knowledge, this is the first study to compare ML algorithms with traditional statistical models to predict a dietary behaviour. We also used nine wellknown ML classification algorithms to conduct analyses. Algorithms showing strong predictive performances will have limited application if execution time is long. In the present case, all algorithms used in this study had relatively short execution time. Despite the relatively small sample size, we included a rather large number of features, which could have allowed ML algorithms to capture non-linear and complex interactions. However, the number of features used may still be considered small according to some standards in the ML field. The extent to which ML classification algorithms outperform traditional statistical models when much larger and complex datasets are used to predict a dietary behavior outcome remains to be investigated.

## Conclusion

ML presents important opportunities for advancing the field of nutritional epidemiology and public health nutrition. However, our results suggest caution regarding the use and added-value of ML classification algorithms to predict diet-related variables and outcomes. Indeed, in the context of predicting adequate VF consumption, ML classification algorithms did not perform better than traditional statistical models. Further research is needed to identify contexts for which ML algorithms are best suited.

## Data Availability Statement

The raw data supporting the conclusions of this article will be made available by the authors, without undue reservation.

## Ethics Statement

The studies involving human participants were reviewed and approved by Université Laval (Ethics Number: 2014-271), Centre hospitalier universitaire de Sherbrooke (Ethics Number: MP-31-2015-997), Montreal Clinical Research Institute (Ethics Number: 2015-02), and Université du Québec à Trois-Rivières (Ethics Number: 15-2009-07.13). The patients/participants provided their written informed consent to participate in this study.

## Author Contributions

MC wrote a first draft of this paper. MC and MAO performed the analyses. ÉC and DB contributed to some of the statistical modeling as well as to generating the data used in this study. SL, JR, MCV, and BL obtained funding for the PREDISE study. FL has contributed to the conceptualization of the analyses and the modeling. BL is the author responsible for this work. All authors contributed to the article and approved the submitted version.

## Funding

MC received a scholarship from the Fonds de recherche du Québec-Santé. The funding organisations were not involved in the writing of this article.

## Conflict of Interest

The authors declare that the research was conducted in the absence of any commercial or financial relationships that could be construed as a potential conflict of interest.

## Publisher's Note

All claims expressed in this article are solely those of the authors and do not necessarily represent those of their affiliated organizations, or those of the publisher, the editors and the reviewers. Any product that may be evaluated in this article, or claim that may be made by its manufacturer, is not guaranteed or endorsed by the publisher.

## Abbreviations

AI: Artificial intelligence
DT: Decision tree
wFFQ: web-based food frequency questionnaire
KNN: K-nearest neighbour
LR: Logistic regression
ML: Machine learning
RF: Random forest
SCM: Set-covering machine
SVM: Support vector machine
VF: Vegetable and fruit.
