# Supplementary material for: Are Machine Learning Algorithms More Accurate in Predicting Vegetable and Fruit Consumption Than Traditional Statistical Models? An Exploratory Analysis
Source: Front Nutr. 2022 Feb 17;9:740898. doi: 10.3389/fnut.2022.740898 (PMC8891134; doi:10.3389/fnut.2022.740898)
Supplement: Supplementary file 1 [file Data_Sheet_1.docx]

***Supplemental Material***

| **Supplemental Table 1**. Description of questionnaires | |  |  |
| --- | --- | --- | --- |
| **Category** | **Name of questionnaire and reference** | **Number of questions** |  |
| **Individual, social and environmental factors** | | |  |
| Attitude and behaviour about diet | Intuitive Eating Scale-2 (1) | 23 |  |
| Sensitivity to reward | Sensitivity to punishment and sensitivity to reward questionnaire (2) | 35 |  |
| Regulation of eating behaviour scale | Regulation of Eating Behaviour Scale (3) | 24 |  |
| Food preferences | Food liking questionnaire (4) | 42 |  |
| Nutrition knowledge | Nutrition knowledge questionnaire (5) | 6 |  |
| Social Factors | Social support for healthy eating questionnaire (6) | 22 |  |
| Accessibility and availability of healthy foods | Perceived food environment (7) | 18 |  |
| Social desirability | Balanced Inventory of Desirable Responding (8) | 40 |  |
| Food behaviour | Three Factor Eating Questionnaire (9) | 51 |  |
| Socioeconomic and demographic factors, eating and lifestyle habits | - | 47 |  |
| Medical | - | 108 |  |
| Physical activity | International Physical Activity Questionnaire (10) | 11 |  |
| **Dietary assessment** | | |  |
| Web-based food frequency questionnaire | Web-FFQ (11) | 136 |  |
| Web-based 24-hour recall | R24W (12-15) | NA |  |

NA: not applicable

| **Supplemental Table 2**. Best hyperparameters for each classification model or algorithm when predicting adequate vegetable and fruit consumption | | |
| --- | --- | --- |
| **Classification algorithm/model** | **Name of hyperparameter** | **Best value** |
| Logistic regression | C | 0.1 |
| Lasso | alpha | 0.019 |
| Decision tree | criterion | gini |
|  | max_depth | 1 |
|  | min_samples_split | 2 |
| Random forest | Criterion | entropy |
|  | Max_depth | 7 |
|  | min_samples_split | 4 |
|  | n_estimators | 60 |
| Set-Covering machine | max_rules | 1 |
|  | model_type | conjunction |
|  | p | 0.85 |
| Support vector machine |  |  |
| *Linear* | C | 0.1 |
|  | gamma | 3 |
| *Polynomial* | C | 1.4 |
|  | degree | 2 |
|  | gamma | 0.001 |
| *RBF* | C | 6 |
|  | gamma | 0.01 |
| *Sigmoid* | C | 1 |
|  | gamma | 0.001 |
| K-nearest neighbor | metric | euclidean |
|  | n_neighbours | 12 |
|  | weights | distance |
| Adaboost | n_estimators | 50 |

**Supplemental Table 3.** Execution time of models and algorithms at random state 42

| **Algorithm** | **Execution time** |
| --- | --- |
| Logistic regression | 7.1 minutes |
| Lasso | 5.4 seconds |
| Decision tree | 7.8 seconds |
| Random forest | 5.5 minutes |
| Set-covering machine | 36.5 seconds |
| Support vector machine (linear) | 1.7 minutes |
| Support vector machine (polynomial) | 4.3 minutes |
| Support vector machine (radial basis) | 1.2 minutes |
| Support vector machine (sigmoid) | 42.2 seconds |
| K-nearest neighbour | 22.2 seconds |
| Adaboost | 11.3 seconds |

**Supplemental Table 4**. Performance metrics of two traditional models and nine machine learning algorithms to predict adequate vegetable and fruit (VF) consumption when other dietary intake features are included in addition to the 2452 features originally included. These are servings of grain products, milk and alternatives, meat and alternatives, as well as components of the Canadian Healthy Eating Index (C-HEI) other than the VF component and the C-HEI score itself.

|  |  | **Algorithms** | | | | | | | | | | |
| --- | --- | --- | --- | --- | --- | --- | --- | --- | --- | --- | --- | --- |
|  | **Performance metric** | **Traditional statistical models (reference)** | | **DT** | **RF** | **SCM** | **SVM** | | | | **KNN** | **Adaboost** |
|  |  | **LR** | **Lasso** |  |  |  | **Linear** | **Polynomial** | **Radial basis** | **Sigmoid** |  |  |
| **Predicting adequate VF consumption (≥5 servings/d)** | **Accuracy TRAIN** | 0.82 | 0.96 | 0.79 | 0.97 | 0.79 | 1.00 | 1.00 | 0.90 | 0.76 | 0.67 | 0.96 |
|  | **Accuracy TEST** | 0.73 | 0.79 | 0.72 | 0.67 | 0.72 | 0.58 | 0.57 | 0.61 | 0.64 | 0.58 | 0.80 |
|  | **AUROC** | 0.74 | 0.87 | 0.72 | 0.67 | 0.72 | 0.58 | 0.57 | 0.61 | 0.64 | 0.58 | 0.80 |
|  | **Positive predictive value^1^** | 0.71 | 0.78 | 0.68 | 0.63 | 0.68 | 0.57 | 0.56 | 0.59 | 0.61 | 0.56 | 0.79 |
|  | **Sensitivity^2^** | 0.79 | 0.81 | 0.80 | 0.80 | 0.80 | 0.64 | 0.65 | 0.71 | 0.78 | 0.69 | 0.81 |
|  | **F1 Score** | 0.75 | 0.79 | 0.74 | 0.71 | 0.74 | 0.60 | 0.60 | 0.65 | 0.68 | 0.62 | 0.80 |

LR, logistic regression; DT, decision tree; RF, random forest; SCM, set-covering machine; SVM, support vector machine; KNN, k-nearest neighbors.

^1^Positive predictive value, also referred to as precision.

^2^Sensitivity, also referred to as recall.

|  | **Supplemental Table 5.** Performance metrics of train and test datasets for each classification model and algorithm to predict adequate vegetable and fruit (VF) consumption based on a different number of features, identified using a feature selection algorithm (see methods). | | | | | | | | | | | | |
| --- | --- | --- | --- | --- | --- | --- | --- | --- | --- | --- | --- | --- | --- |
|  |  |  | Algorithms | | | | | | | | | | |
|  | Performance metrics | Number of features | Traditional statistical models (reference) | | DT | RF | SCM | SVM | | | | KNN | Adaboost |
|  |  |  | LR | Lasso |  |  |  | Linear | Polynomial | Radial basis | Sigmoid |  |  |
| Predicting adequate VF consumption (≥5 servings/d) | Accuracy TRAIN | 5 features | 0.56 | 0.56 | 0.56 | 0.67 | 0.56 | 0.56 | 0.56 | 0.56 | 0.56 | 0.67 | 0.61 |
|  |  | 10 features | 0.63 | 0.63 | 0.64 | 0.66 | 0.64 | 0.62 | 0.67 | 0.65 | 0.60 | 0.65 | 0.64 |
|  |  | 50 features | 0.65 | 0.66 | 0.66 | 0.76 | 0.62 | 0.66 | 0.70 | 0.66 | 0.66 | 0.99 | 0.70 |
|  |  | All features | 0.75 | 0.76 | 0.65 | 0.91 | 0.65 | 1.00 | 1.00 | 0.83 | 0.76 | 0.67 | 0.79 |
|  | Accuracy TEST | 5 features | 0.55 | 0.51 | 0.51 | 0.57 | 0.51 | 0.51 | 0.51 | 0.51 | 0.51 | 0.57 | 0.55 |
|  |  | 10 features | 0.62 | 0.62 | 0.60 | 0.60 | 0.60 | 0.60 | 0.59 | 0.58 | 0.61 | 0.54 | 0.60 |
|  |  | 50 features | 0.58 | 0.56 | 0.60 | 0.60 | 0.59 | 0.57 | 0.58 | 0.59 | 0.60 | 0.61 | 0.57 |
|  |  | All features | 0.63 | 0.62 | 0.57 | 0.63 | 0.57 | 0.55 | 0.56 | 0.64 | 0.64 | 0.60 | 0.58 |
|  | AUROC | 5 features | 0.55 | 0.54 | 0.51 | 0.57 | 0.51 | 0.51 | 0.51 | 0.51 | 0.51 | 0.56 | 0.55 |
|  |  | 10 features | 0.62 | 0.63 | 0.60 | 0.60 | 0.60 | 0.61 | 0.59 | 0.58 | 0.61 | 0.54 | 0.60 |
|  |  | 50 features | 0.58 | 0.64 | 0.60 | 0.60 | 0.59 | 0.57 | 0.58 | 0.59 | 0.60 | 0.61 | 0.57 |
|  |  | All features | 0.63 | 0.65 | 0.57 | 0.63 | 0.57 | 0.55 | 0.56 | 0.64 | 0.64 | 0.60 | 0.58 |
|  | Positive predictive value^1^ | 5 features | 0.54 | 0.50 | 0.50 | 0.55 | 0.50 | 0.50 | 0.50 | 0.50 | 0.50 | 0.58 | 0.54 |
|  |  | 10 features | 0.59 | 0.59 | 0.59 | 0.58 | 0.59 | 0.58 | 0.57 | 0.55 | 0.58 | 0.54 | 0.59 |
|  |  | 50 features | 0.56 | 0.55 | 0.58 | 0.58 | 0.58 | 0.55 | 0.57 | 0.57 | 0.57 | 0.58 | 0.56 |
|  |  | All features | 0.60 | 0.60 | 0.57 | 0.59 | 0.57 | 0.55 | 0.54 | 0.61 | 0.61 | 0.58 | 0.57 |
|  | Sensitivity^2^ | 5 features | 0.67 | 0.70 | 0.70 | 0.75 | 0.70 | 0.70 | 0.70 | 0.70 | 0.70 | 0.43 | 0.63 |
|  |  | 10 features | 0.76 | 0.79 | 0.67 | 0.68 | 0.67 | 0.76 | 0.67 | 0.79 | 0.76 | 0.54 | 0.67 |
|  |  | 50 features | 0.65 | 0.66 | 0.66 | 0.69 | 0.67 | 0.65 | 0.64 | 0.75 | 0.76 | 0.80 | 0.62 |
|  |  | All features | 0.72 | 0.72 | 0.58 | 0.77 | 0.58 | 0.58 | 0.66 | 0.78 | 0.78 | 0.69 | 0.61 |
|  | F1 score | 5 features | 0.59 | 0.59 | 0.59 | 0.63 | 0.59 | 0.59 | 0.59 | 0.59 | 0.59 | 0.49 | 0.58 |
|  |  | 10 features | 0.67 | 0.67 | 0.63 | 0.63 | 0.63 | 0.67 | 0.62 | 0.65 | 0.66 | 0.54 | 0.63 |
|  |  | 50 features | 0.60 | 0.60 | 0.62 | 0.63 | 0.62 | 0.60 | 0.60 | 0.64 | 0.65 | 0.67 | 0.59 |
|  |  | All features | 0.66 | 0.65 | 0.57 | 0.67 | 0.57 | 0.56 | 0.60 | 0.68 | 0.68 | 0.63 | 0.59 |

LR, logistic regression; DT, decision tree; RF, random forest; SCM, set-covering machine; SVM, support vector machine; KNN, k-nearest neighbors.

^1^Positive predictive value, also referred to as precision.

^2^Sensitivity, also referred to as recall

**Supplemental Figure 1.** Comparing accuracy of models predicting adequate vegetable and fruit consumption (≥ 5 servings/d) when using non-normalized versus normalized data from continuous features. LR: logistic regression; DT: decision tree; RF: random forest; SCM: set-covering machine; SVM: support vector machine; KNN: k-nearest neighbour.

**
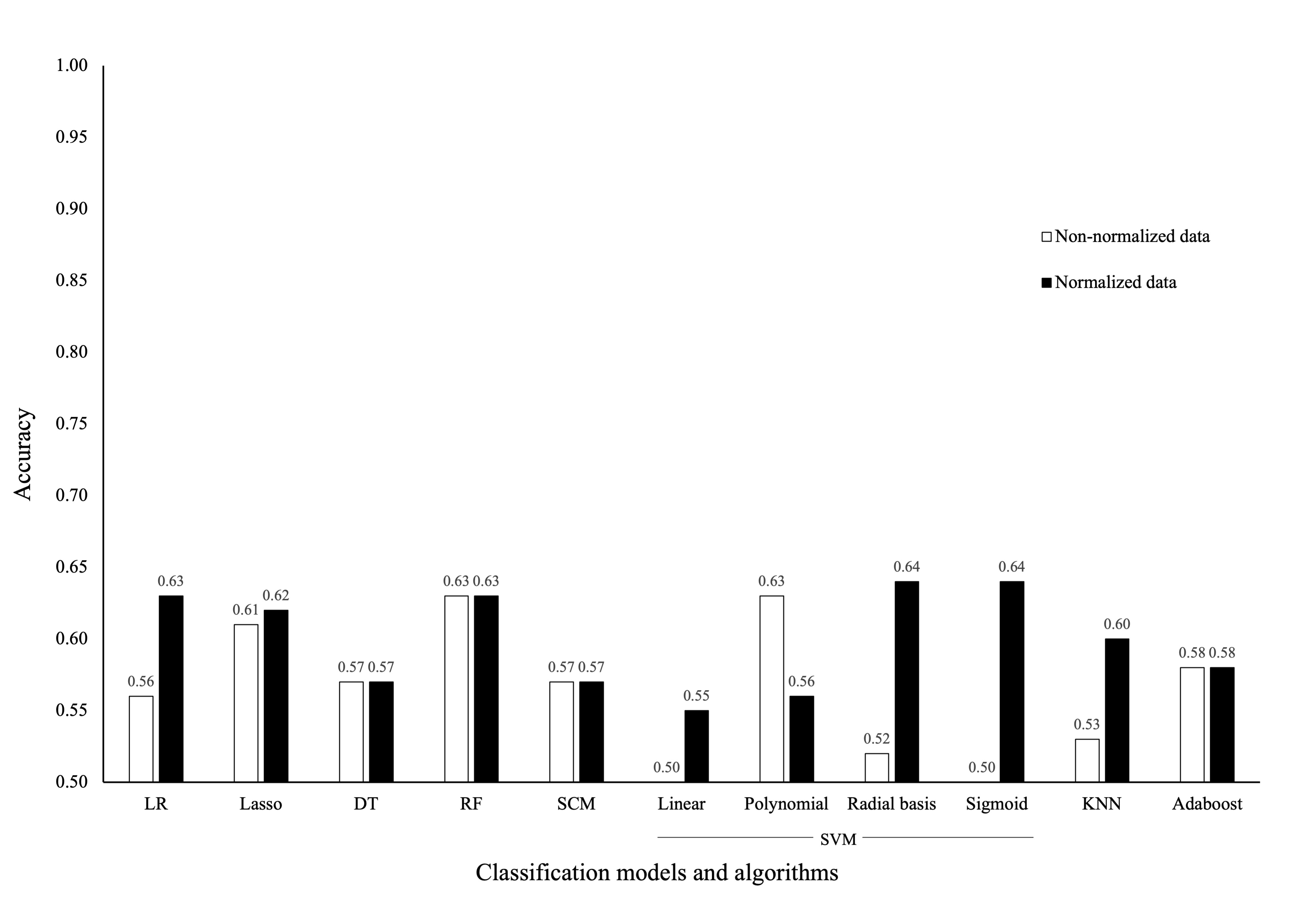
**

**Supplemental Figure 2.** Comparing accuracy of models predicting adequate vegetable and fruit consumption (≥ 5 servings/d) based on a different number of features identified using a feature selection algorithm (see methods). LR: logistic regression; DT: decision tree; RF: random forest; SCM: set-covering machine; SVM: support vector machine; KNN: k-nearest neighbour.

**
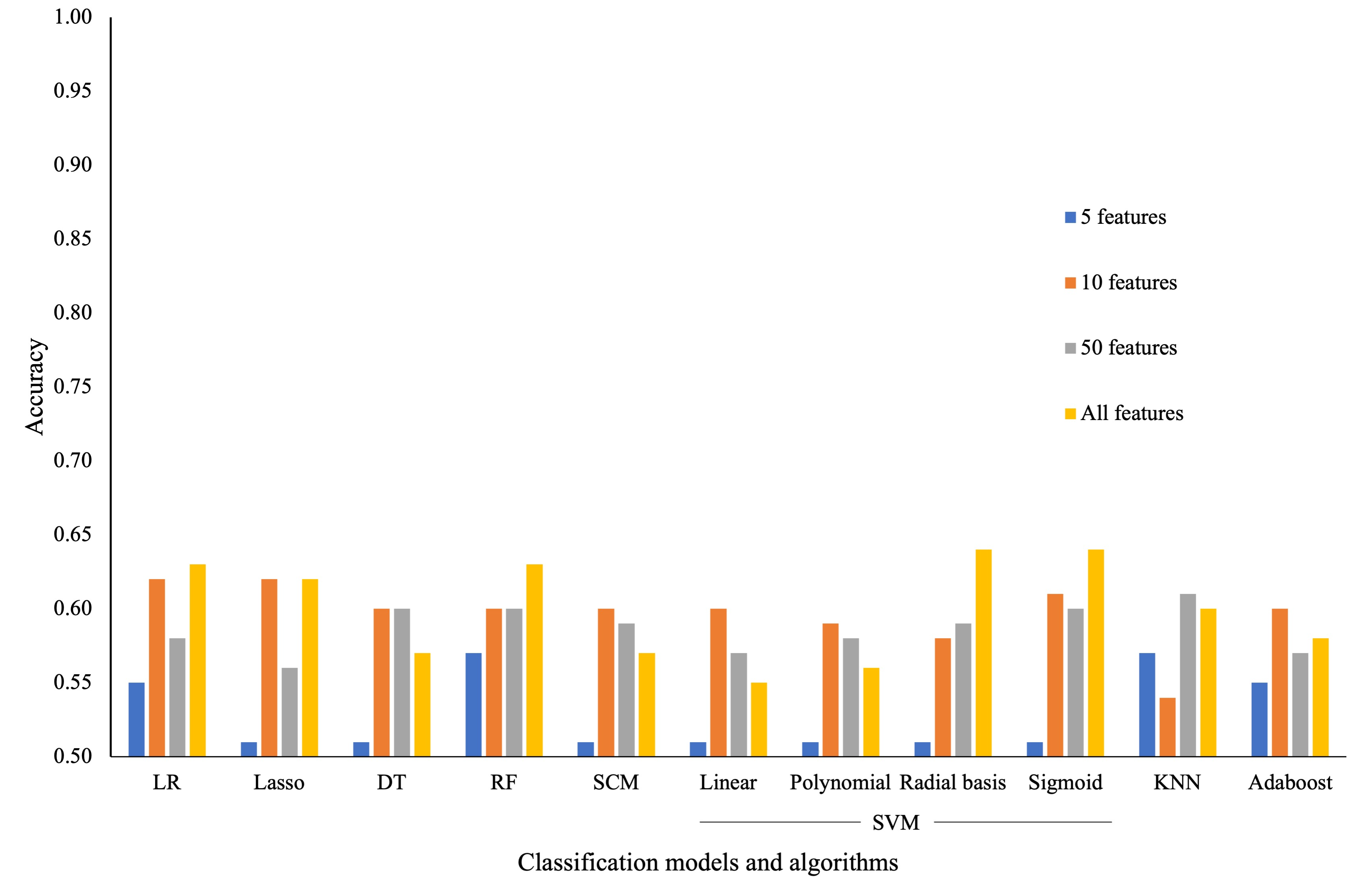
**

**References**

1. Carbonneau E, Carbonneau N, Lamarche B, Provencher V, Bégin C, Bradette-Laplante M, et al. Validation of a French-Canadian adaptation of the Intuitive Eating Scale-2 for the adult population. Appetite. 2016;105:37-45.

2. Lardi C, Billieux J, d’Acremont M, Linden MVd. A French adaptation of a short version of the Sensitivity to Punishment and Sensitivity to Reward Questionnaire (SPSRQ). Personality and Individual Differences. 2008;45(8):722-5.

3. Pelletier LG, Dion SC, Slovinec-D'Angelo M, Reid R. Why Do You Regulate What You Eat? Relationships Between Forms of Regulation, Eating Behaviors, Sustained Dietary Behavior Change, and Psychological Adjustment. Motivation and Emotion. 2004;28(3):245-77.

4. Carbonneau E, Bradette-Laplante M, Lamarche B, Provencher V, Bégin C, Robitaille J, et al. Development and Validation of the Food Liking Questionnaire in a French-Canadian Population. Nutrients. 2017;9(12):1337.

5. Bradette-Laplante M, Carbonneau É, Provencher V, Bégin C, Robitaille J, Desroches S, et al. Development and validation of a nutrition knowledge questionnaire for a Canadian population. Public Health Nutrition. 2017;20(7):1184-92.

6. Carbonneau E, Bradette-Laplante M, Lamarche B, Provencher V, Bégin C, Robitaille J, et al. Social support for healthy eating: development and validation of a questionnaire for the French-Canadian population. Public Health Nutrition. 2018:1-7.

7. Carbonneau E, Robitaille J, Lamarche B, Corneau L, Lemieux S. Development and validation of the Perceived Food Environment Questionnaire in a French-Canadian population. Public Health Nutrition. 2017;20(11):1914-20.

8. Paulhus D. Measurement and Control of Response Bias. In: Robinson JP, Shaver, P.R., Wrightsman, L. S. , editor. Measures of personality and social psychological attitudes. San Diego, CA: Academic Press; 1991. p. 17-59.

9. Stunkard AJ, Messick S. The three-factor eating questionnaire to measure dietary restraint, disinhibition and hunger. Journal of psychosomatic research. 1985;29(1):71-83.

10. Craig CL, Marshall AL, Sjöström M, Bauman AE, Booth ML, Ainsworth BE, et al. International physical activity questionnaire: 12-country reliability and validity. Medicine and science in sports and exercise. 2003;35(8):1381-95.

11. Labonté MÈ, Cyr A, Baril-Gravel L, Royer MM, Lamarche B. Validity and reproducibility of a web-based, self-administered food frequency questionnaire. European Journal of Clinical Nutrition. 2012;66(2):166-73.

12. Jacques S, Lemieux S, Lamarche B, Laramée C, Corneau L, Lapointe A, et al. Development of a Web-Based 24-h Dietary Recall for a French-Canadian Population. Nutrients. 2016;8(11):724.

13. Lafrenière J, Lamarche B, Laramée C, Robitaille J, Lemieux S. Validation of a newly automated web-based 24-hour dietary recall using fully controlled feeding studies. BMC Nutrition. 2017;3(1).

14. Lafrenière J, Laramée C, Robitaille J, Lamarche B, Lemieux S. Assessing the relative validity of a new, web-based, self-administered 24 h dietary recall in a French-Canadian population. Public Health Nutrition. 2018;21(15):2744-52.

15. Lafreniere J, Laramee C, Robitaille J, Lamarche B, Lemieux S. Relative validity of a web-based, self-administered, 24-h dietary recall to evaluate adherence to Canadian dietary guidelines. Nutrition. 2019;57:252-6.
